# A Moderated Mediation Model of Maternal Perinatal Stress, Anxiety, Infant Perceptions and Breastfeeding

**DOI:** 10.3390/nu11122981

**Published:** 2019-12-06

**Authors:** Jessica P. Riedstra, Nicki L. Aubuchon-Endsley

**Affiliations:** Department of Psychology, Idaho State University, Pocatello, ID 83209, USA; aubunick@isu.edu

**Keywords:** perinatal, maternal stress, maternal anxiety, infant crying, breastfeeding

## Abstract

This study examined a moderated mediation model of relations among maternal perinatal stress/anxiety, breastfeeding difficulties (mediator), misperceptions of infant crying (moderator), and maternal breastfeeding duration to understand risk factors for early breastfeeding termination. It was hypothesized that more breastfeeding difficulties would mediate the relation between greater prenatal stress/anxiety and shorter breastfeeding duration, and that perceptions of response to infant crying as spoiling would moderate the relation between more breastfeeding difficulties and reduced breastfeeding duration. Additionally, it was hypothesized that participants who breastfed through 6 months would demonstrate less postnatal stress/anxiety and there would be a positive relation between fewer breastfeeding difficulties and less postnatal stress/anxiety through 6 months. Participants included 94 expectant mothers at 33–37 weeks gestation and 6 months (±2 weeks) postpartum. Greater prenatal anxiety was associated with shorter breastfeeding duration. Results presented are the first to document negative relations between prenatal (as opposed to postnatal) anxiety and breastfeeding duration (as opposed to frequency or other indicators) in a U.S. sample. Future studies should seek to replicate findings in a more diverse sample and compare findings from clinical and non-clinical samples. Studies may also wish to explore the effects of anxiety prevention/intervention on breastfeeding duration.

## 1. Introduction

Despite efforts to improve breastfeeding rates, about half of mothers discontinue breastfeeding by 6 months, and two-thirds by 12 months [1]. Breastfed children and mothers who breastfeed benefit from improved physical and psychological health and maternal perinatal stress and anxiety may impact breastfeeding behaviors [2,3,4,5,6,7,8,9,10,11]. About 10% of pregnant women and 13% of women who recently gave birth have a mental disorder (most commonly depression and anxiety) [12]. While perinatal depression is heavily researched, stress and anxiety receive considerably less attention and remain less understood [13,14,15,16,17]. Furthermore, limited research has examined these psychological experiences among medically underserved perinatal women (e.g., those with limited access to physical and mental health services). 

Feelings of stress (i.e., reaction to a stressor) and anxiety (i.e., emotion not necessarily tied to a specific event or stressor) can be defined as two separate, though similar, constructs [18]. Literature suggests that women may experience perinatal stress and/or anxiety; therefore, it is important to measure them separately. For this project, subjective measures of stress and anxiety were utilized given the practical utility for perinatal health providers. Regarding anxiety, worldwide estimates suggest 7%–60% of women experience high levels of general anxiety during pregnancy and 5%–33% in the postpartum period. Furthermore, 3%–39% of women experience an anxiety disorder, as do 4%–20% of postpartum women [15]. In addition, 17% of Idaho women experience some form of anxiety 3 months prior to pregnancy [19]. Research regarding the prevalence of psychosocial stress during pregnancy is limited, and suggests that during the antenatal period, 6% of women reported high stress, 78% low to moderate stress, and 16% no stress [20]. In 2010, 70% of women reported a stressful life event within the year prior to their infant’s birth [21]. With regard to Idaho, 21% of women described experiencing high prenatal stress (i.e., three or more stressful life events in the 12 months prior to delivery) [19]. Therefore, women in this region may experience more pregnancy stressful life events than the national average, making perinatal samples from these understudied regions important to consider. Many of these women also have poorer access to healthcare, thereby enhancing the potential impact and outcomes of stress-related difficulties during pregnancy [22]. 

### 1.1. Breastfeeding Difficulties

Numerous research findings support relations between stress or anxiety and lower breastfeeding rates [23,24,25,26,27,28,29,30,31]. Breastfeeding difficulties also may play a role (e.g., mediation) in breastfeeding behaviors. Early in the postpartum period, breastfeeding concerns or perceptions, like difficulty latching or low milk supply, are a significant source of maternal stress [32]. The Centers for Disease Control (CDC) Pregnancy Risk Assessment Monitoring System (PRAMS) is perhaps the most comprehensive and widely used assessment of U.S. women’s experiences during and 2–6 months after pregnancy, including breastfeeding. It is utilized to examine difficulties initiating and continuing breastfeeding, including: Difficulties for mother, infant, and with time management, among others [33]. Women discontinue breastfeeding due to physical pain and discomfort (e.g., cracked nipples, biting, and scratching); uncertainty of milk supply and latching issues; personal, professional, social, and physical difficulties; beliefs regarding infant preferences; and lack of confidence [9,11,34,35]. Several breastfeeding difficulties may influence breastfeeding initiation, frequency, and duration, however, the relation between stress and anxiety and breastfeeding difficulties remains unexplored. 

### 1.2. Infant Crying 

Considering the potential relation between breastfeeding difficulties and behaviors, perceptions of infant crying may serve as a moderating factor. Crying can be disruptive and challenging for families, including experiencing guilt, stress, frustration, social isolation, and strained relationships [36]. Many mothers use breastfeeding as a method to console crying infants, and breastfeeding to comfort is a strong predictor of longer partial breastfeeding duration [37]. However, women who perceive responding to their infant’s crying cues with breastfeeding as spoiling may experience a more robust relation between increased breastfeeding difficulties and decreased breastfeeding behaviors [38,39]. The compounded effect of breastfeeding difficulties and a decreased response to infant crying with breastfeeding may serve to decrease breastfeeding duration, such that women may avoid difficulties and the risk of spoiling their infants by removing feeding to console. Therefore, maternal perceptions of spoiling an infant via breastfeeding to console crying behavior were investigated in the present study. 

Taken together, little, if any, research has focused on women from medically underserved, U.S. samples wherein breastfeeding resources and support may be limited. Few studies have examined both anxiety and stress, and their impact on specific breastfeeding behaviors during the prenatal and postpartum periods. To our knowledge, no research has evaluated mechanisms by which perinatal stress, anxiety, breastfeeding difficulties, perceptions of responding to infant crying, and breastfeeding behaviors may be related. Additionally, research to date has demonstrated variability in when and how peripartum stress and/or anxiety is measured. Thus, the present study used psychometrically sound measures at two time points to explore whether prenatal (i.e., during third trimester) or postnatal (i.e., up to 6 months postpartum) stress or anxiety is more predictive of breastfeeding behavior. The study also sought to examine the role of breastfeeding difficulties as a potential mediator of relations between prenatal stress and anxiety and breastfeeding duration, and perceptions of infant crying as a moderator between breastfeeding difficulties and duration. Additionally, the study sought to examine univariate relations between postnatal maternal stress, anxiety, and breastfeeding difficulties, relevant covariates, and breastfeeding duration. Understanding risk factors that contribute to decreased breastfeeding behavior may prove beneficial for infants and mothers, which may assist in prevention/intervention research. The following models and hypotheses were used to fill these gaps in the extant literature.

### 1.3. Proposed Models and Hypotheses

Given the relation between breastfeeding difficulties and duration, it is plausible that breastfeeding difficulties mediate the relation between stress and anxiety and breastfeeding duration [34,35]. Furthermore, a mother’s perception of responding to infant crying with breastfeeding as spoiling may interact with breastfeeding difficulties to further negatively impact breastfeeding duration. Therefore, high scores on measures of maternal misperceptions of infant crying may moderate, or strengthen, the mediated relation between anxiety and stress, breastfeeding difficulties, and breastfeeding duration. Given these predicted relations, two moderated mediation models were proposed: Hypothesis 1 consisted of a moderated mediation model with prenatal stress as the predictor, breastfeeding difficulties as the mediator, perceptions of infant crying as spoiling as the moderator along the b path, and breastfeeding duration through 6 months as the outcome variable. Hypothesis 2 proposed the same model, however the predictor was prenatal anxiety. To examine the impact of breastfeeding experiences on postnatal stress and anxiety, two further relations were hypothesized. Specifically, it was hypothesized that participants who breastfed through 6 months postpartum would demonstrate lower levels of postnatal stress (Hypothesis 3) and anxiety (Hypothesis 4) than those who discontinued prior to 6 months. We also hypothesized that among women who breastfeed through 6 months, there would be a positive relation between fewer breastfeeding difficulties and lower levels of postnatal stress (Hypothesis 5) and anxiety (Hypothesis 6). As breastfeeding difficulties appear related to shorter breastfeeding duration, women who continue breastfeeding at 6 months may experience fewer difficulties and report lower levels of stress and anxiety. 

## 2. Methods 

### 2.1. Participants

**Figure 1:**
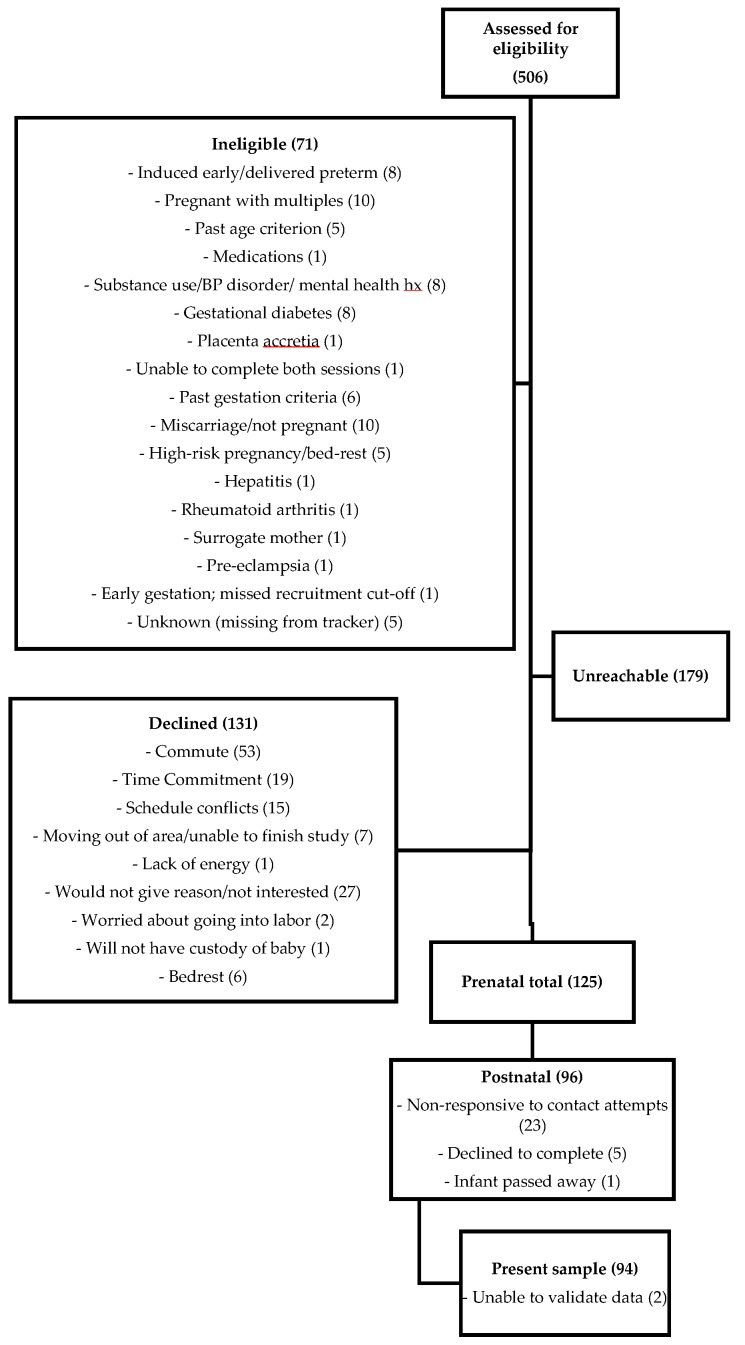
Response rate throughout recruitment and each data collection time point. Bolded numbers in parentheses indicate total sample size at each stage and non-bolded numbers in parentheses indicate the number of participants who withdrew for the reason listed.

Data were collected as part of a larger longitudinal research project examining maternal health and infant development. The project was approved by the Idaho State University Human Subjects Committee (protocol 4191, entitled “Infant Development and Healthy Outcomes in Mothers Study”). Data were collected for 125 prenatal participants and 96 participants at 6 months postpartum (See Figure 1). 

### 2.2. Recruitment 

Participants were recruited throughout southeastern Idaho and screened for eligibility via brief phone calls. During the third trimester, prospective participants met individually with a trained research assistant (RA) and completed written informed consent, if eligible. Exclusion criteria included mothers with: More than one baby; certain health conditions (e.g., gestational diabetes, pre-eclampsia, and toxemia) that could impact endocrine functioning; certain behavioral or physical health diagnoses/symptoms (e.g., Schizophrenia, Bipolar Disorder, HIV, and AIDS); or chronic use of recreational substances (e.g., marijuana, or cocaine), medications from FDA categories D and X with documented detrimental fetal effects, or alcohol (>40 drinks) during pregnancy. Mothers had to be at least 18 years of age to provide consent, and 35 years of age or younger given that infertility, chromosomal abnormalities, miscarriage, stillbirth, and multiple pregnancies occur at greater rates after age 35 and all of these factors are related to other exclusion criteria [40]. Participants were recruited from medical centers (e.g., hospitals, clinics), family programs and service centers, childcare facilities, local businesses, schools/university, libraries, recreational centers, the local tribal reservation, community organizations and professionals (e.g., doulas, La Leche League, midwives), and through a variety of mediums (e.g., newsletters, social media). 

### 2.3. Quantitative Analyses

Based on Fritz and MacKinnon’s mediation model simulations, a sample size of 71 is required to achieve a power of 0.80 in a mediation model assessed via bias-corrected bootstrapping with medium effect sizes (*d* = 0.39) for a and b paths [41]. Similarly, a priori power analyses utilizing *GPower* suggest that a sample size of 85 is sufficient for the most complex analyses tested in Hypothesis 1 and 2 [41,42]. In *GPower*, a least squares linear multiple regression with three predictors and one outcome variable and up to three covariates was used. This included a medium effect size (*f*^2^ = 0.15) for *R* squared change in steps 1 and 2 after covariates then predictors are added into the model. Analyses also included a two-tailed *p*-value of 0.05 and power of 0.80. Medium effects size parameters were used based upon prior research findings on maternal affect and breastfeeding behaviors [14]. Two participants’ data on the Perinatal Anxiety Screening Scale (PASS) were deemed to be likely inaccurate due to extremely low scores, which indicated that participants did not attend to measure items. Therefore, they were removed from the dataset, resulting in a final sample size of 94. The PROCESS macro v2.16, Model 14 was used to test moderated mediation Hypotheses 1 and 2 [43]. The macro uses bias-corrected bootstrapping to assess statistical significance of direct and indirect effects, which maximizes power and is robust against Type II error and violations of normality. These methods require a smaller sample size to attain sufficient power for the conditional indirect effect with recent simulation studies supporting that sample sizes <100 have power ≥0.80 with a and b paths of medium effect size [44]. The macro quantifies the effect of V on the indirect effect of X on Y through M, with significance determined via bias-corrected confidence intervals [43]. Independent samples *t*-tests were used to test Hypotheses 3 and 4, and Pearson’s product–moment correlations for Hypotheses 5 and 6. Power analyses utilizing *GPower* suggested a sample size of 82 to achieve a medium effect (i.e., 0.3) and a power of 0.80 [42]. Prenatal and postnatal PASS scores were positively skewed and transformed using a natural log function.

### 2.4. Measures

#### 2.4.1. Stress 

The Perceived Stress Scale (PSS) is a 14-item self-report measure developed to assess the degree to which respondents find their lives “unpredictable, uncontrollable, and overloading,” or components central to experiencing stress [45] (p. 387). In two college and one smoking cessation sample, the PSS demonstrated Cronbach’s α = 0.84−0.86, test-retest reliability after 2 days of 0.85, and test-retest reliability after 6 days of 0.55. The College Student Life-Event Schedule and PSS correlated between 0.17–0.49 in all samples [46]. The PSS-10 (α = 0.74) and PSS-4 (α = 0.79) have demonstrated good reliability in pregnancy samples [47] and a recent, large (*n* = 2847) validation study suggested that the PSS-14 has similar factor structure, internal consistency, and convergent and divergent validity than the PSS-10 in two prenatal samples [48]. The PSS demonstrated good reliability at prenatal and 6-month postnatal time points in the current sample (Cronbach’s *a* = 0.80 and 0.78, respectively). 

#### 2.4.2. Anxiety

The PASS is a 31-item self-report questionnaire developed to screen for a broad range of anxiety symptoms in perinatal women [49]. The PASS total score is statistically significantly correlated with the Depression Anxiety Stress Scale anxiety (Pearson’s product–moment *r* = 0.78, *p* < 0.01) and stress (Pearson’s product–moment *r* = 0.81, *p* < 0.01) scales, anxiety scale of the Edinburg Postnatal Depression Scale (Pearson’s product–moment *r* = 0.74, *p* < 0.01) and the State–Trait Anxiety Inventory (STAI; State Pearson’s product–moment *r* = 0.75, *p* < 0.01; Trait Pearson’s product–moment *r* = 0.83, *p* < 0.01)), which support the measure’s convergent validity [50,51]. A correlation between PASS scores in subsamples of antenatal and postnatal women (*n* = 35) was 0.74. Internal consistency of the total PASS score in the present sample was high at prenatal (*a* = 0.95) and postnatal (*a* = 0.93) time points. 

#### 2.4.3. Breastfeeding difficulties

Breastfeeding difficulties were measured as part of the 6-Month Infant Dietary Questionnaire, a measure created utilizing similar item content from the PRAMS. The 7-item self-report questionnaire measures infant feeding behaviors since birth, including formula feeding, breastfeeding, consuming solid foods, and difficulties breastfeeding. Breastfeeding difficulties were measured with a yes/no question asking whether difficulties have been present, at any time and with any frequency, during breastfeeding. Mothers answering “yes” were asked to specify which difficulties they experienced including maternal-related difficulties (i.e., expressing milk, soreness, or fatigue), difficulties an infant may experience (i.e., latching, sucking, not obtaining enough milk, or not interested in feeding), difficulties with time management (i.e., returning to work and unable to pump), difficulties with insufficient environment (i.e., discomfort feeding in public or around others at home), or others (e.g., open-ended question). Difficulties were quantified as the number endorsed (ranging from 0 to 10 or more). Frequency of difficulties were provided as percentages (See Results). 

#### 2.4.4. Infant crying behavior 

The Infant Crying Questionnaire-Revised (ICQ-R) is a 43-item self-report measure of maternal perceptions of infant crying [52]. The postnatal version includes 20 items that assess mothers’ perceptions of their babies’ crying behavior. The ICQ-R consists of five scales, and the present study utilized the Spoiling scale as an index of maternal perceptions of response to infant crying as spoiling. The Spoiling scale has demonstrated internal consistency of Cronbach’s α = 0.70 [52]. Scores for the three items related to infant spoiling are totaled. The Spoiling scale in the present sample demonstrated Cronbach’s *a* = 0.76. 

#### 2.4.5. Breastfeeding duration 

Breastfeeding duration was measured as part of the 6-Month Infant Dietary Questionnaire [53] via a single open-ended item asking participant how many months, weeks, or days they breastfed. All data were quantified as days. 

### 2.5. Procedures 

The prenatal visit included an interview regarding participants’ current and past pregnancy-related information, brief health history, and sociodemographic characteristics (socioeconomic status, ethnicity, race, age), and electronic self-report questionnaires including the PSS and PASS. Participants were reimbursed $30 for completing the prenatal session. A mental health resource list was provided if participants endorsed any critical items or reported experiencing distress. RAs scheduled participant’s 6-month postnatal session 1 month after their due date and sent reminders to ensure sessions occurred as scheduled. During the 6-month session, mothers completed interviews regarding their and their baby’s health, the 6-Month Infant Dietary Questionnaire, and the following electronic self-report measures: PSS, PASS, and ICQ-R postpartum version. 

## 3. Results

### 3.1. Sample Characteristics 

Data were collected from adult mothers (M_AGE_ = 27.29 years, SD_AGE_ = 4.02 years) during the third trimester (M_GESTATION_ = 34.25 weeks, SD_GESTATION_ = 1.22 weeks), and 6 months postpartum (M_AGE_ = 6.05 months, SD_AGE_ = 2.13 months). The largest percentage of participants identified as White/Caucasian (94%), married (84%), and members of the Church of Jesus Christ of Latter-day Saints (LDS; 62%), with annual household incomes between $50,000–$74,999 (29%), having completed a standard college or university degree (i.e., BS/BA; 38%), employed (60%), and having birthed one other child (30%; see Table 1). Additionally, 14% qualified as living in a rural area [22]. Chi-square tests of independence revealed that there were no significant differences in race (χ^2^(2) = 3.119, *p* = 0.210), marital status (χ^2^(1) = 1.407, *p* = 0.236), employment status (χ^2^(1) = 3.185, *p* = 0.074) or education status (χ^2^(20) = 12.951, *p* = 0.879) between participants who chose to return for the 6 month session and those who did not. Independent samples *t*-tests also revealed no differences in income (t(217) = −0.289, *p* = 0.773), prenatal stress (t(123) = −0.924, *p* = 0.358), prenatal anxiety (t(123) = −1.289, *p* = 0.200), and parity (t(123) = 0.187, *p* = 0.187) between time points. Religious preference data were only collected at 6 months. 

### 3.2. Correlations

Pearson correlations between primary predictor and outcome variables (See Table 2) supported significant relations between stress and anxiety at both time points. None of the potential covariates (number of maternal prenatal, labor/delivery, or postnatal health conditions, infant physical health conditions through 6 months of age, socioeconomic status (SES) measured by the Hollingshead Four Factor Index of Social Status, and parity) significantly correlated with predictor and outcome variables and were not included in moderated mediation models [54]. SES was related to postnatal stress (*r* = −0.312, *p* = 0.002) and anxiety (*r* = −0.275, *p* = 0.007), colic was related to postnatal stress (*r* = 0.215, *p* = 0.04), and postnatal maternal illness was related to postnatal anxiety (*r* = 0.257, *p* = 0.05). Therefore, these variables were controlled for in follow-up analyses for Hypotheses 3 and 4. 

### 3.3. Descriptive Statistics

Sixty five percent of participants reported breastfeeding at 6 months postpartum, with 86% having initiated breastfeeding since giving birth. The average PSS score was 19.59 (SD = 6.65) prenatally and 20.23 (SD = 5.96) at the 6-month postnatal visit, out of 56 points. Participants’ average PASS score was 16.73 (SD = 11.99) prenatally and 14.46 (SD = 9.45) postnatally out of 93 points. Both PASS average scores fall in the minimal anxiety range. On average, participants ICQ-R Spoiling score was 5.62 (SD = 2.10) out of 15. Participants reported an average of 0.52 (SD = 0.96) difficulties with breastfeeding. Specifically, 19% endorsed difficulties for themselves, 17% endorsed difficulties for their babies, 11% endorsed difficulties with time management, 2% endorsed an insufficient environment, and 3% noted other difficulties, although their responses were found to be iterations of the previously mentioned answer choices. Participants reported an average of 0.83 (SD = 0.94) prenatal maternal illnesses, 0.64 (SD = 0.80) postnatal maternal illnesses, 1.62 (SD = 0.49) complications during labor or delivery, and 4.83 (SD = 2.09) infant illnesses from birth to 6 months postpartum. The average duration of breastfeeding through 6 months was 138 days (SD = 69.13 days) or 4.4 months.

**Hypothesis** **1.** *The moderated mediation model proposed in Hypothesis 1 was not statistically significant (See Table 3).*

**Hypothesis** **2.** *The moderated mediation model proposed in Hypothesis 2 was also not statistically significant (See Table 3). However, the model with prenatal anxiety as the predictor revealed that prenatal anxiety significantly predicted breastfeeding duration (b = −25.253, t(93) = −2.325, SE = 10.860, p = 0.022).*

An independent samples *t*-test was conducted to determine the difference in breastfeeding duration between the 14 participants (approximately 15% of mothers) who were at or above the prenatal PASS cut-off of 26 and those below. All 96 participants were used, as natural log transformations were only necessary for postnatal PASS scores. The two groups were statistically significantly different (t(94) = −3.90, *p* < 0.001, *d* = 1.044), with mothers who were above the clinical cut score for prenatal anxiety breastfeeding 72.58 days or 2.5 months less in duration. Of the mothers with clinically elevated prenatal anxiety, only 4 (29%) breastfed through 6 months, while 56 of the 82 (68%) mothers who did not have clinically elevated prenatal anxiety continued breastfeeding at 6 months. Due to small sample sizes, results should be replicated before drawing strong conclusions.

**Hypothesis** **3.** *We conducted an independent samples t-test to compare women who breastfed through 6 months to women who did not on postnatal stress (Hypothesis 3). Results suggests no significant differences in postnatal stress (t(92) = −0.711, p = 0.479). As follow-up analyses, we examined the relation between breastfeeding duration as a continuous variable, and postnatal stress and anxiety, while controlling for covariates. An analysis of covariance (ANCOVA) suggested that the relationship between stress and breastfeeding duration was not statistically significant (F(13) = 0.753, p = 0.706), though SES was significantly related to postnatal stress (F(1) = 7.865, p = 0.006).*

**Hypothesis** **4.** *We conducted an independent samples t-test to compare women who breastfed through 6 months to women who did not on postnatal anxiety (Hypothesis 4). Results suggests no significant differences in postnatal anxiety (t(92) = −1.369, p = 0.174). An additional ANCOVA suggested that the relationship between greater postnatal anxiety and longer breastfeeding duration was statistically significant (F(13) = 2.385, p = 0.009), even after controlling for postnatal maternal illness and SES.*

**Hypothesis** **5.** *Utilizing a two-tailed Pearson correlation among women who continued to breastfeed through 6 months postpartum, breastfeeding difficulties were not significantly related to postnatal stress (Hypothesis 5, r = −0.156, p = 0.114).*

**Hypothesis** **6.** *An additional two-tailed Pearson correlation also suggested that breastfeeding difficulties were not significantly related to postnatal anxiety among women who continued to breastfeed through 6 months postpartum, (Hypothesis 6, r = −0.137, p = 0.144).*

## 4. Discussion

The purpose of the present project was to investigate a novel mediator and moderator in the relation between prenatal and postnatal anxiety and stress and breastfeeding duration among a medically underserved sample. While none of the moderated mediation models were significant, results indicated that greater prenatal anxiety was significantly related to shorter breastfeeding duration. This finding replicates previous literature in non-U.S. samples suggesting that high levels of trait anxiety in early and mid-pregnancy were associated with decreased breastfeeding rates [25]. Furthermore, many women who experience anxiety during pregnancy breastfeed for less than 6 months [26]. Researchers speculate that poor psychosocial health during pregnancy may be an indicator of poorer overall health, which may influence breastfeeding behaviors [25,26]. 

Prior literature suggests that prenatal anxiety predicts shorter exclusive breastfeeding at 1 month postpartum, but only prenatal depression is related to breastfeeding duration [55]. A literature review revealed mixed findings regarding prenatal anxiety and breastfeeding behaviors [56]. While there are no associations between prenatal anxiety and breastfeeding initiation, a relation does exist between high levels of prenatal anxiety and a decrease in breastfeeding intention and exclusivity. The relation between prenatal anxiety and breastfeeding is still poorly understood and necessitates further research [56]. Researchers report possible explanations for this relation, including that trait anxiety may interfere with oxytocin release, which stimulates the milk-ejection reflex [56]. State anxiety may result in heightened levels of cortisol and glucose, potentially decreasing milk volume and resulting in decreased breastfeeding duration. Additionally, mothers who experience prenatal anxiety about parenting may avoid challenging parenting behaviors, such as breastfeeding, leading to decreased maternal-infant interaction and decreased breastfeeding. 

Few, if any studies have examined and found that various forms of prenatal anxiety, including state, trait, and perinatal, influence breastfeeding duration. Thus, the present project provides novel and important findings regarding correlates of breastfeeding duration. Future research should investigate whether prenatal state, strait, or perinatal anxiety is most related to breastfeeding duration. While the present study provides further support for the relation between heightened prenatal anxiety and decreased breastfeeding duration, the interactive biopsychosocial mechanisms by which this relation exists remains elusive and should be further explored (e.g., stress hormone systems, immune functioning, maternal-infant bonding, and sociocultural understanding and support for breastfeeding). Our analyses also suggest a positive relation between postnatal anxiety and breastfeeding duration, which further indicates the importance of continuing to examine mechanisms of action (e.g., mediating role of breastfeeding difficulties) for these complex and poorly understood relations. Additionally, the positive relation between increased SES and increased postnatal stress may be due to factors like occupation, which warrants investigation. 

Correlation analyses suggest that stress and anxiety are related within and over time. Moreover, prenatal and postnatal anxiety were also significantly related to perceptions of infant spoiling. As previous literature suggests, excessive infant crying can be challenging for mothers, and this may lead to heightened feelings of stress and anxiety [36]. If mothers misperceive their response to infant cries with breastfeeding as spoiling, they may decrease breastfeeding behaviors [38,39]. Subsequently, mothers’ stress and anxiety may increase if they notice a decrease in their milk supply due to decreased feeding behavior, or due to infant crying behavior that they are not addressing. Additional studies are needed to further investigate these relations.

Our study variables were grounded in strong theory and largely assessed by psychometrically sound measures, and conditional process analysis allowed for a robust and multivariate examination of prenatal stress and anxiety. However, the present study also included limitations, including the way in which we measured breastfeeding difficulties/duration and perinatal stress, homogenous sample demographics (e.g., White), relatively lower power for Hypotheses 5 and 6, and a correlational design. Possible future directions include creating comprehensive and well-validated measures of breastfeeding behaviors, difficulties, and psychosocial experiences, tracking breastfeeding duration and anxiety at multiple time points and beyond 6 months postpartum, examining other breastfeeding outcomes (e.g., initiation or frequency), studying more heterogeneous samples, exploring additional risk and reliance factors, and examining perceptions of infant crying more broadly. Future research should continue to explore possible explanations for decreased breastfeeding duration among women with psychological distress, as these relations are still not fully understood. Focus in each of these areas will lead to a richer understanding of maternal experiences in the perinatal period, informing future intervention and policy. 

### Practical Implications

The current study’s findings hold important implications. Although our sample was a relatively low-risk, healthy community sample, we still found a significant relation between greater prenatal anxiety and shorter breastfeeding duration. Furthermore, even minimal levels of prenatal anxiety are related to breastfeeding duration, thus pregnant women with low levels of anxiety may benefit from screening and intervention. Future prevention and intervention research should focus on the nature of maternal prenatal anxiety, including women who experience relatively low levels of anxiety as well as clinical samples. Understanding the characteristics of prenatal anxiety may help inform more specific targets for psychosocial prevention, intervention, and support. 

Follow-up analysis using the PASS cut-off score of 26, demonstrated that mothers who experienced clinically significant levels of prenatal anxiety on average breastfed for 72.58 days, approximately 2.5 months, less than mothers without clinically significant levels of prenatal anxiety (i.e., 148 days or almost 5 months). The magnitude of this effect was quantified by a large Cohen’s *d* effect size as well, indicating a strong statistical effect (*d* = 1.044). A review of the literature suggests that even a 2.5-month difference in breastfeeding duration is clinically meaningful, including numerous implications in maternal and infant physical health (e.g., infant risk of infectious diseases) [57,58]. Of note, only 4 of the 14 mothers who were elevated on the prenatal PASS breastfed through 6 months, while 56 of the 82 mothers who did not have clinically elevated prenatal anxiety continued breastfeeding at 6 months. This suggests that the effects of prenatal anxiety on breastfeeding duration may play a robust and critical role within the first few months, such that this is a time period when breastfeeding difficulties should be assessed in greater detail and breastfeeding resources, support, and interventions may be most efficacious. As the two groups in the present analyses were not proportional (i.e., only 14 mothers met the threshold for clinically significant prenatal anxiety), findings should be replicated with larger sample sizes. Nonetheless, these findings indicate that maternal prenatal anxiety may be clinically significantly related to breastfeeding duration, and subsequently, health outcomes for mother-infant dyads. While the present study examined several possible covariates in relation to these variables, breastfeeding duration and PASS scores were related even after controlling for maternal illness and SES. Future studies should consider examining the role of social support among other factors that may influence broad relations between maternal affect and breastfeeding outcomes. 

## 5. Conclusions

The present project sought to better understand the relations among perinatal stress, anxiety, breastfeeding difficulties, perceptions of responding to infant crying, and breastfeeding duration in an underserved sample. Previous cross-cultural research has demonstrated some relations among these variables, however there is inconsistency in these findings. Furthermore, this is the first study to our knowledge to examine these variables collectively through moderated mediation models. The present project also fills a gap in the literature by exploring the unique experiences of U.S. women, specifically in Idaho. Study results revealed a statistically and clinically significant relation between clinical levels of prenatal anxiety and decreased breastfeeding duration, highlighting that even low levels of prenatal anxiety should not be overlooked in research or clinical settings. 

## Figures and Tables

**Table 1:** Sample sociodemographic description.

Race (Categories Not Mutually Exclusive)	*n*	%
White/Caucasian	88	94
Black/African American	2	2
Native Hawaiian or other Pacific Islander	2	2
American Indian/Alaska Native	1	1
Hispanic/Latino	13	14
Asian	1	1
Other	5	5
**Relationship Status**		
Single/never married	9	10
Married	79	84
Divorced	1	1
Committed relationship	3	3
Engaged	2	2
**Employment Status**		
Employed	56	60
Not currently working	38	40
**Highest Degree of Education**		
Partial high school	2	2
High school	13	14
Partial college	33	35
Standard college or university	36	38
Graduate training with a degree	10	11
**Income ($)**		
<5000	1	1
5000–9000	2	2
10,000–19,000	14	15
20,000–29,000	16	17
30,000–39,000	12	13
40,000–49,000	9	10
50,000–74,999	27	29
75,000–99,999	7	7
≥100,000	6	6
**Religious Preference**		
Agnostic	3	3
Assembly of God	2	2
Atheist	2	2
Baptist	2	2
Catholic	4	4
Lutheran	2	2
Methodist	1	1
Church of Jesus Christ of Latter-day Saints	58	62
Non-denominational	10	11
Pentecostal	1	1
Presbyterian	1	1
Other	12	13
Prefer not to answer	9	10
**Parity**		
No other child	39	41
One other child	28	30
Two other children	12	13
Three other children	6	6
Four other children	3	3
5 other children	5	5
6 other children	1	1

Note. *n* = 94. Categories for race and religious preference were not mutually exclusive.

**Table 2:** Correlations among primary study variables.

	PASS (Prenatal)	PSS (Prenatal)	PASS (6 M)	PSS (6 M)	Bf diff.	Spoiling	Duration
PASS (prenatal)	1						
PSS (prenatal)	0.686 **	1					
PASS (6 M)	0.608 **	0.506 **	1				
PSS (6 M)	0.483 **	0.578 **	0.487 **	1			
Bf difficulties	0.036	0.019	0.109	0.027	1		
Spoiling	0.218 *	0.178	0.222 *	0.169	−0.028	1	
Duration	−0.258 *	−0.121	−0.155	−0.130	−0.105	−0.102	1

Note. PASS = Perinatal Anxiety Screening Scale; PSS = Perceived Stress Scale; 6 M = postnatal 6-month time point; Bf = breastfeeding; Spoiling = response to infant crying with breastfeeding as spoiling; Duration = breastfeeding duration. * *p* < 0.05. ** *p* < 0.01.

**Table 3:** Prenatal moderated mediation findings.

	F	*R* ^2^	S/A → bf diff	Bf Difficulties → Duration	S/A → Duration	Interaction (Bf Difficulties × Spoiling)
			*b/SE*	*b/SE*	*b/SE*	*b/SE*
Stress	0.747	0.033	0.003/0.015	−10.416/24.829	−1.068/1.103	0.503/4.201
Anxiety	1.889	0.078	0.052/0.149	−6.633/24.299	−25.253 */10.860	0.069/4.109

Note. Bf = breastfeeding; Spoiling = response to infant crying with breastfeeding as spoiling; Duration = breastfeeding duration; SE = standard error; S/A = stress/anxiety. Arrows indicate direction of prediction. * *p* < 0.05.
